# Regulating the Rheology of Drilling Fluids Under High-Temperature Conditions with Hydrophobically Associating Polymers

**DOI:** 10.3390/polym18070859

**Published:** 2026-03-31

**Authors:** Xuyang Yao, Kaihe Lv, Jing He, Tao Ren, Cheng Ye

**Affiliations:** 1 School of Petroleum Engineering, China University of Petroleum (East China), Qingdao 266580, China; yaoxuyang2019@petrochina.com.cn; 2 Oil Production Technology Research Institute of PetroChina Xinjiang Oilfield Company, Karamay 834000, China; jinghe@petrochina.com.cn (J.H.); rent2022@petrochina.com.cn (T.R.); sunych66@petrochina.com.cn (C.Y.)

**Keywords:** water-based drilling fluid, viscosity enhancer, polymer, hydrophobic association, temperature and salt tolerance

## Abstract

As global oil and gas exploration extends to deep and ultra-deep formations, high-temperature and high-salt environments have become major challenges for drilling fluid viscosifiers. In this study, a hydrophobic associative polymer viscosifier, HATA, was synthesized via free-radical copolymerization using acrylamide (AM), 2-acrylamido-2-methylpropanesulfonic acid (AMPS), sodium styrene sulfonate (SSS), and stearyl methacrylate (SMA) as monomers, and its structure was systematically characterized, while its performance and action mechanism in a 4 wt% bentonite base slurry were evaluated. The results show that the base slurry modified with 3 wt% HATA maintains an apparent viscosity retention ratio of 69.20% following 16 h of hot rolling at 180 °C, with an API filtration loss of only 7.2 mL, and its HTHP filtration loss is 73.72% lower than that of the blank bentonite slurry system; this viscosifier sustains effective viscosity and yield point of the drilling fluid system at 200 °C and in 36 wt% NaCl brine. HATA achieves viscosity enhancement and filtration control by regulating surface charges of bentonite particles, constructing stable three-dimensional networks, and stabilizing clay hydration layers, thus presenting a high-performance viscosifier formulation for high-temperature and high-salinity water-based drilling fluids with important theoretical and engineering application values.

## 1. Introduction

With the continuous advancement of global oil and gas exploration and development toward deep and ultra-deep formations, drilling operations are increasingly challenged by more complex geological environments. Extreme downhole conditions (high temperature, high pressure, and high salinity) place more stringent demands on the operational safety and drilling efficiency of these processes [1,2,3,4]. During drilling, drilling fluids perform critical functions: wellbore stabilization, drill cuttings transportation, formation pressure balancing, bottom-hole cleaning, and drill string lubrication [5,6]. Owing to their low cost and environmental compatibility, water-based drilling fluids (WBDFs) have become the most widely used fluid systems. However, in deep and ultra-deep formations, WBDFs exhibit significant rheological degradation and a sharp increase in filtration loss. In severe instances, such degradation leads to critical drilling hazards, including wellbore collapse and differential sticking [7,8]. Therefore, developing key additives capable of maintaining superior rheological and filtration properties under high-temperature and high-salinity conditions is of great significance for ensuring safe and efficient drilling operations in deep formations.

Viscosifiers are critical additives for regulating the rheological properties of WBDFs. They confer shear-thinning behavior to minimize circulation friction loss and provide a sufficiently high low-shear-rate viscosity (LSRV) for solid particle suspension (cuttings, bridging agents) and efficient hole cleaning [9,10]. Currently, polymeric viscosifiers used in WBDFs can be generally categorized into two types: naturally modified polymers and synthetic polymers [11,12,13]. Common naturally modified polymeric viscosifiers include hydrophobically modified hydroxyethyl cellulose (HMHEC), xanthan gum derivatives, and starch-based polymers, which offer environmental compatibility and broad availability [14,15]. However, their molecular structures contain glycosidic and ether linkages that undergo thermal degradation at elevated temperatures [10,16]. Additionally, in high-salinity environments, electrostatic shielding causes their molecular chains to adopt a compact coiled conformation, leading to severe viscosity reduction and limited applicability in deep/ultra-deep formations [17,18,19]. Due to their highly tunable molecular structures, synthetic polymers have attracted significant attention as potential viscosifiers for high-temperature and high-salinity environments [19,20,21]. Researchers have improved the thermal and salt tolerance of synthetic polymers via rational monomer selection and molecular structure design. For instance, Xie et al. synthesized a hydrophobically associating polymer viscosifier (SDKP) by copolymerizing N-vinylcaprolactam and 2-acrylamido-2-methylpropanesulfonic acid sodium, with divinylbenzene serving as a crosslinker [22]. When added at 1.5 wt%, SDKP maintained stable rheological properties after 160 °C hot rolling aging in a 5 wt% NaCl solution; its apparent viscosity (AV), plastic viscosity (PV), and yield point (YP) decreased by only 3.5 mPa·s, 3 mPa·s, and 0.5 Pa, respectively. The API and HTHP filtrate losses were significantly lower than those of the commercial rheology modifier HE300. However, this study did not evaluate rheological behavior above 160 °C or under high-salinity conditions. Similarly, Wang synthesized a novel amphiphilic copolymer (SPXFJ) containing amide and sulfonic functional groups via free-radical polymerization, which demonstrated excellent thermo-thickening properties after aging at 180 °C [23]. Results from high-temperature aging and HTHP simulation tests indicated that WBDFs containing SPXFJ exhibited remarkable anti-settling performance with no barite sedimentation observed at 180 °C, 100 MPa, and a fluid density of 2.0 g/cm^3^. Nevertheless, the influence of salinity on the rheological behavior of SPXFJ-based drilling fluids was not investigated. Zhang et al. also developed a zwitterionic hydrophobically associating polymer (PAADDC) by copolymerizing five monomers via free-radical polymerization for use in WBDFs [22]. However, rheological evaluation revealed a significant decline in performance when the aging temperature increased to 180 °C, with AV, PV, and YP dropping to 7.5 mPa·s, 6.5 mPa·s, and 1.0 Pa, respectively.

To overcome the inadequate thermal and salt tolerance of commercial WBDF viscosifiers in deep-reservoir conditions, we synthesized a novel hydrophobically associating polymeric viscosifier (HATA) in this work. The polymer was prepared by copolymerizing acrylamide, 2-acrylamido-2-methylpropanesulfonic acid, sodium styrene sulfonate, and octadecyl methacrylate. The molecular structure, thermal stability, molecular weight distribution, and microstructure were characterized using Fourier transform infrared spectroscopy, thermogravimetric analysis, gel permeation chromatography, and cryogenic scanning electron microscopy, respectively. Rheological properties, including shear thinning behavior, thixotropy, and yield point, were examined using a HAAKE rotational rheometer. A comprehensive evaluation was conducted to investigate the effects of polymer dosage, temperature, and salinity on viscosity enhancement, thermal and salt tolerance, and filtration control performance in a 4 wt% base mud system. Furthermore, zeta potential analysis, particle size distribution (PSD) measurements, and filter cake scanning electron microscopy (SEM) were used to elucidate the interaction mechanism between HATA and bentonite clay particles. The findings of this work provide a promising approach for developing key additives that ensure the stable performance of WBDFs in deep, high-temperature, and high-salinity drilling environments.

## 2. Materials and Methods

### 2.1. Materials

The chemicals used in this study include acrylamide (AM, 99 wt%), 2-acrylamido-2-methylpropanesulfonic acid (AMPS, 99 wt%), sodium chloride (NaCl, 99.5%), potassium chloride (KCl, 99.5%), and anhydrous sodium carbonate (Na_2_CO_3_, 99.5%), all purchased from Aladdin Biochemical Technology Co., Ltd. (Shanghai, China). Additionally, sodium styrene sulfonate (SSS, 99 wt%), stearyl methacrylate (SMA, 99 wt%), and NaOH (analytical purity, prepared as a 35 wt% solution) were supplied by Macklin Biochemical Technology Co., Ltd. (Shanghai, China). Ammonium persulfate (analytical purity) was obtained from Sinopharm Chemical Reagent Co., Ltd. (Shanghai, China). Bentonite was procured from Huaian County Tengfei Bentonite Development Co., Ltd. (Zhangjiakou, China).

The viscosifier DSP-2 was sourced from Shandong Deshunyuan Petroleum Technology Co., Ltd. (Dongying, China). DSP-2 is synthesized via multi-step polymerization from acrylamide, acrylic acid, 2-acryloyloxybutyl sulfonic acid, epichlorohydrin, and a novel cationic monomer with a cyclic structure. Its active content is higher than 75%, and the number-average molecular weight (Mn) is approximately 1,300,000.

### 2.2. Synthesis of Copolymer

**Figure 1:**
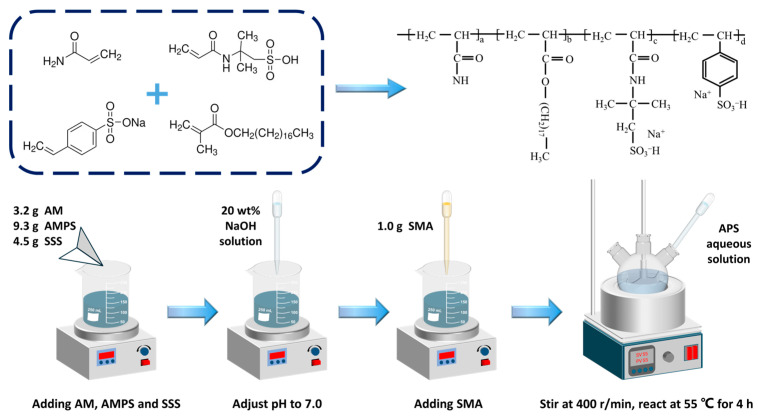
Schematic preparation process of HATA.

A high-temperature-resistant viscosifier for water-based drilling fluids was prepared via free radical polymerization. As shown in Figure 1, 100 g of deionized water was added to a 250 mL beaker. Under high-speed magnetic stirring, 3.2 g of AM, 9.3 g of AMPS, and 4.5 g of SSS were sequentially added to the beaker and stirred to dissolve the three polymerization monomers in the aqueous phase. Subsequently, the 20 wt% NaOH aqueous solution was used to adjust the pH of the system to 7, followed by adding 1.0 g of SMA into the aqueous phase and stirring at high speed for 20 min. Next, the above system was transferred to a three-necked flask equipped with a nitrogen gas device, a thermometer, and a stirring device. The flask was heated in a water bath set at 55 °C. Finally, nitrogen gas was introduced into the flask, 0.02 g of the initiator (NH_4_)_2_S_2_O_8_ was dissolved in 10 mL of deionized water, and the initiator solution was added dropwise into the flask via a constant pressure funnel. The reaction was carried out at 55 °C for 4 h with a stirring speed of 400 r/min. After the reaction, the viscous liquid in the flask was placed in an oven at 70 °C, dried to constant weight, and ground to obtain the hydrophobically associating, high-temperature-resistant viscosifier. The molecular structure of the viscosifier is shown in Figure 1, denoted as HATA.

### 2.3. Characterization and Analysis

HATA was dried to constant weight in a drying oven. The Fourier transform infrared (FT-IR) spectrum of the viscosifier HATA was determined using a Fourier transform infrared spectrometer (IRTRacer-1000, Shimadzu, Kyoto, Japan). The samples were prepared by the tablet pressing method (the mass ratio of HATA to potassium bromide was approximately 1:100). The specific test parameters were as follows: the number of scans was 30, and the wavenumber range was from 400 to 4000 cm^−1^.

HATA was dried to constant weight in a drying oven. The thermal stability of HATA was determined using a simultaneous thermal analyzer (STA 409 PC, Netzsch GmbH, Selb, Germany). The specific test parameters were as follows: temperature range of 40 °C to 600 °C, heating rate of 10 °C/min, and nitrogen gas as the protective gas.

The relative molecular mass of HATA was determined by a gel permeation chromatograph (Malvern Viscotek 3580, Malvern Panalytical Ltd., Malvern, Worcestershire, UK). HATA was dissolved in ultrapure water to prepare an aqueous HATA solution with a mass fraction of 0.5%. This solution was filtered through a 0.22 μm filter membrane. A 10–100 μL aliquot of the filtered sample was drawn with a syringe and injected into the instrument’s injection valve, with precautions taken to avoid air bubble entrainment. The instrument’s analysis program was initiated, and the flow rate and running time (30–60 min) were set. The system was then allowed to complete chromatogram generation.

### 2.4. Aqueous Rheological Evaluation

A HAAKE MARS rotational rheometer (Thermo Fisher Scientific, Karlsruhe, Germany) was used to characterize the rheological properties of aqueous HATA, DSP-2 solutions at various concentrations, and 2 wt% HATA/DSP-2 solutions in saline media.

To determine the microstructure reconstruction time, 1.0 wt% and 2.0 wt% samples were pre-sheared at 300 s^−1^ for 100 s to reach a steady state. Dynamic time sweep tests were then performed using a plate fixture at room temperature with 0.1 Pa constant stress and 1 Hz frequency to monitor the evolution of G′ and G′′. All samples were statically equilibrated for the corresponding time to ensure consistent initial microstructures and reliable, repeatable results.

Strain sweeps were first conducted to determine the linear viscoelastic range using a plate fixture at room temperature. Frequency sweep tests from 0.1 to 100 rad/s were then performed to obtain G′ and G′′ and analyze the frequency-dependent viscoelastic behavior.

Steady shear rheological tests from 0.01 to 1000 s^−1^ were carried out with a coaxial cylinder fixture at room temperature for 2 wt% HATA and DSP-2 solutions in salt-free, 5 wt% KCl, and 25 wt% NaCl media to investigate shear-thinning behavior and salt tolerance.

Thixotropy and shear recovery tests were also performed using a coaxial cylinder fixture. Thixotropic behavior was evaluated via a shear rate cycle of 0 → 1000 → 0 s^−1^ to obtain hysteresis loop areas. For shear recovery, samples were pre-sheared at 1000 s^−1^, and viscosity recovery was recorded over 0–180 s under static conditions to quantify recovery efficiency.

### 2.5. Preparation of Drilling Fluid

Take 400 mL of distilled water and place it in a high-speed mixing cup. Add 16 g of bentonite and 0.56 g of anhydrous sodium carbonate sequentially at a stirring speed of 8000 rpm, and continue stirring for 20 min. During the stirring process, pause twice to scrape off residues adhering to the cup wall and the stirring blades. Subsequently, seal the mixture and allow it to cure at room temperature for 24 h to obtain the water-based drilling fluid sample.

### 2.6. Performance Evaluation of Drilling Fluids

HATA was added to both the drilling fluid and the saltwater drilling fluid. The mixtures were stirred at 8000 rpm for 20 min at room temperature to obtain the drilling fluid samples for testing. The rheological parameters of the drilling fluids were measured using a ZNN-D6 six-speed rotational viscometer (Tongchun Petroleum Instrument Co., Ltd., Qingdao, China). The performance was compared with that of the viscosifier DSP-2. The drilling fluid and saltwater drilling fluid were placed in aging cells and hot-rolled in a roller oven (Tongchun Petroleum Instrument Co., Ltd., Qingdao, China) at the test temperature for 16 h. After retrieval and cooling to room temperature, their rheological parameters were measured again. Based on the readings at 600 rpm and 300 rpm, the apparent viscosity (AV), plastic viscosity (PV), and yield point (YP) were calculated using the following formulas:

AV = 0.5 × θ600 (mPa·s)(1)

PV = θ600 − θ300 (mPa·s)(2)

YP = 0.511 × (θ300 − PV) (Pa)(3)

The API fluid loss (FL_API_) of the drilling fluid was measured at ambient temperature under a pressure of 100 psi for 30 min using an SD6A medium-pressure filter press (Tongchun Petroleum Instrument Co., Ltd., Qingdao, China), in accordance with American Petroleum Institute (API) standards. The prepared drilling fluid was placed in aging cells and hot-rolled in a roller oven (Qingdao Tongchun Petroleum Instrument Co., Ltd., China) at the test temperature for 16 h. After retrieval and cooling to room temperature, the API fluid loss was measured again. Furthermore, the high-temperature, high-pressure (HTHP) fluid loss (FL_HTHP_) of the aged drilling fluid was tested using a GGS71-B HTHP filter press (Qingdao Tongchun Petroleum Instrument Co., Ltd., China). The test duration was 30 min, and the test pressure differential was 3.5 MPa. All the above API fluid loss and HTHP fluid loss tests were repeated three times for each group, and the average values were taken as the final test results.

### 2.7. Thickening Mechanism Analysis

The drilling fluid samples were diluted 1000-fold using deionized water, and zeta potential measurements were conducted using a nanoparticle analyzer (Zetasizer Nano Z, Malvern Instruments Ltd., Malvern, UK). Each sample was tested three times, with the average value recorded.

The adsorption capacity of HATA on bentonite was measured using a TOC-L total organic carbon analyzer (Shimadzu Corporation, Kyoto, Japan). Each sample was tested in triplicate, and the average value was reported.

The particle size distribution of the drilling fluid was analyzed using a Mastersizer-3000 E particle size analyzer (Malvern, UK) before and after 16 h of aging at 180 °C.

The filter cake obtained from the API fluid loss test was subjected to freeze-drying. The microstructure of the freeze-dried filter cake was then observed using an EVO-15/LS scanning electron microscope (SEM; Carl Zeiss AG, Oberkochen, Germany).

The colloidal microstructure of drilling fluid samples was observed by cryo-scanning electron microscopy. Samples were dropped onto the sample stage, rapidly frozen in liquid nitrogen slurry for 60 s, and sputter-coated with gold prior to observation. The cold stage temperature was −120 °C, and the accelerating voltage was 5 kV. The cryo-SEM observations were performed on a Zeiss Crossbeam 550 (Carl Zeiss AG, Oberkochen, Germany) equipped with a cryo-transfer system (Gatan Alto 2500, Gatan Inc., Pleasanton, CA, USA).

## 3. Results

### 3.1. Characterization and Analysis of HATA

#### 3.1.1. Infrared Spectrogram Analysis

**Figure 2:**
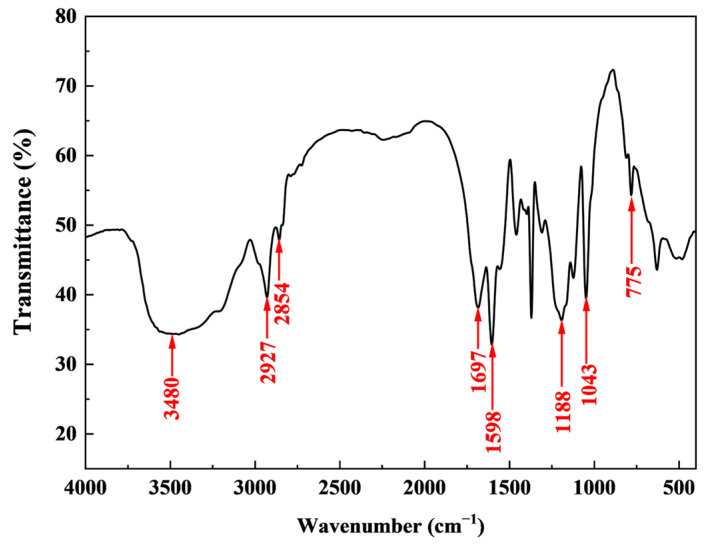
FT-IR spectrum of HATA.

The FT-IR spectrum of HATA is shown in Figure 2. All characteristic functional groups of the copolymerized monomers were detected, confirming the successful synthesis of the target product HATA. The absorption peak at 3480 cm^−1^ was caused by the stretching vibration of N-H in the amide group (-CONH_2_) (from AM and AMPS) [24]. The absorption peaks at 2927 cm^−1^ and 2854 cm^−1^ were caused by the stretching vibrations of -CH_3_ and -CH_2_ (from AMPS, SMA, etc.) [25]. The absorption peak at 1697 cm^−1^ was attributed to the stretching vibration of -C=O (from AM and AMPS) [26]. The absorption peak at 1598 cm^−1^ was the skeletal vibration peak of the benzene ring (from SSS) [27]. The absorption peak at 1188 cm^−1^ was caused by the S=O stretching vibration of -SO_3_^−^ (from SSS) [28].

#### 3.1.2. TGA Analysis

**Figure 3:**
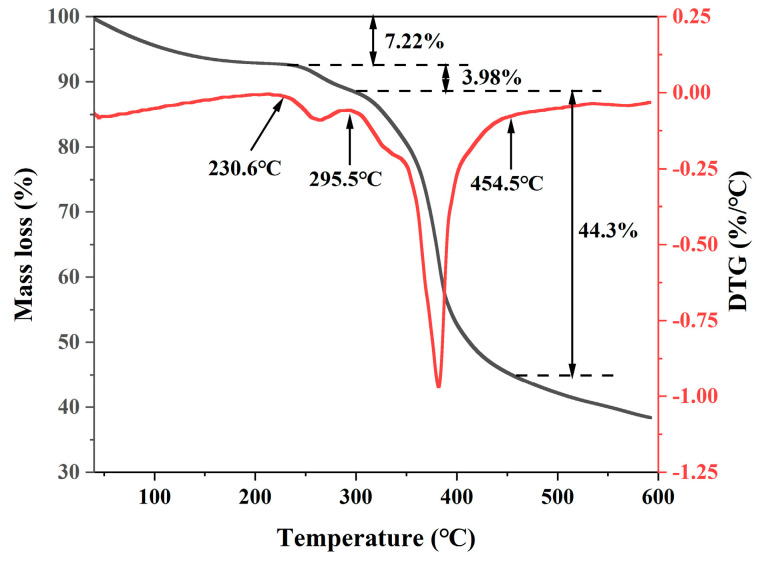
TGA curve of HATA.

The thermogravimetric (TG) curve of HATA (mass variation with temperature) is shown in Figure 3. Figure 3 shows that HATA undergoes three distinct stages of mass loss during thermal treatment: 40–230.6 °C, 230.6–295.5 °C, and 295.5–454.5 °C. In the 40–230.6 °C stage, the mass loss rate of HATA was 7.22%. This mass loss was mainly caused by the evaporation of free water adsorbed on the surface of HATA and bound water absorbed by hydrophilic groups [29]. In the 230.6–295.5 °C stage, the mass loss rate of HATA was 3.98%, which was primarily attributed to the thermal degradation of side chains [30,31]. In the 295.5–454.5 °C stage, the DTG curve of HATA exhibited a rapid decrease and a subsequent rapid increase, indicating that HATA underwent rapid thermal degradation. The mass loss rate in this stage reached 44.3%, mainly resulting from the high-temperature degradation and carbonization of the main chain of HATA [32,33]. When the temperature exceeded 454.5 °C, the mass of HATA continued to decrease, demonstrating that HATA is undergoing continuous high-temperature degradation. In summary, the initial thermal decomposition temperature of HATA reached 230.6 °C, indicating that it still maintained good stability at high temperatures and was suitable for high-temperature drilling processes.

#### 3.1.3. GPC Analysis

**Figure 4:**
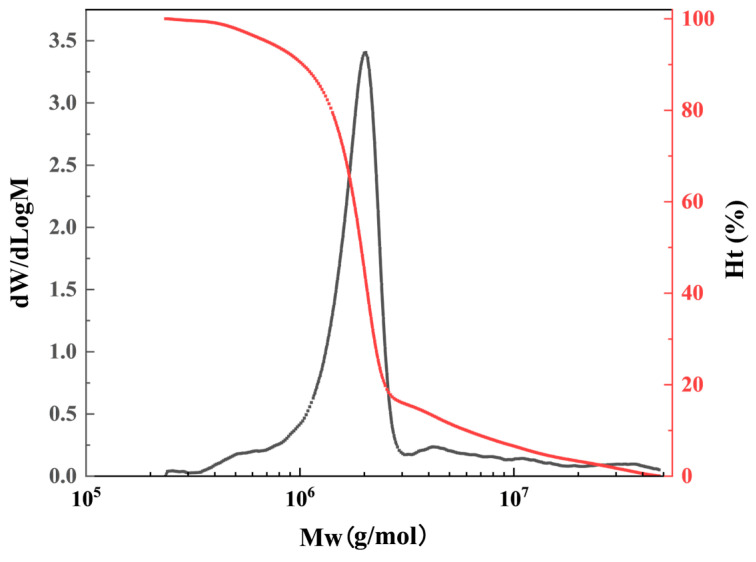
GPC curve of HATA.

The relative molecular weight distribution of HATA is shown in Figure 4 and Table 1. HATA exhibits a relatively narrow molecular weight distribution, with a weight-average molecular weight (Mw) of 3,449,076, a number-average molecular weight (Mn) of 1,658,370, a Z-average molecular weight (Mz) of 13,512,573, and a polydispersity index (PD) of 2.08. Both Mw and Mn of HATA exceed 1,200,000, indicating that the viscosifier HATA is a polymer with an ultra-high molecular weight surpassing one million, which meets the requirements of its molecular structure design. The incorporation of large molecular weight HATA into drilling fluids plays a positive role in enhancing their viscosity.

### 3.2. Rheological Testing of HATA in Water

**Figure 5:**
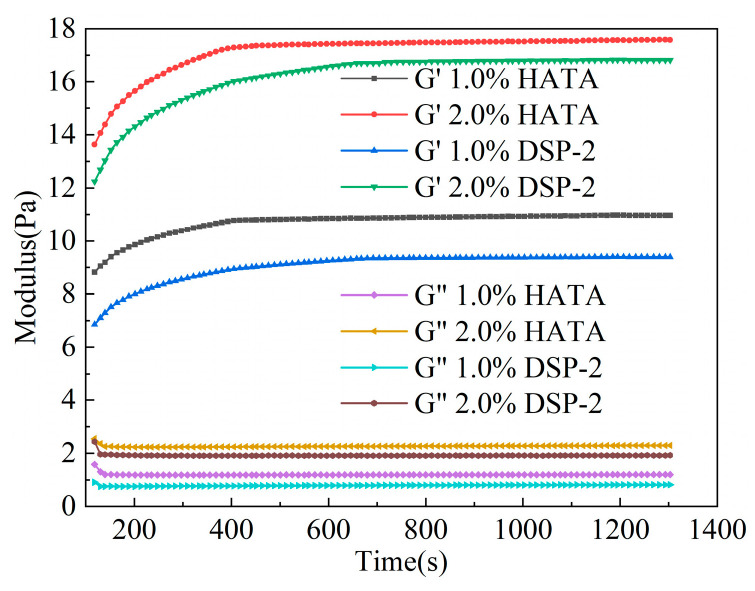
Time oscillation sweep: Variation curves of G′ and G″ with time.

Time-sweep rheological curves of aqueous HATA and DSP-2 solutions with different concentrations are shown in Figure 5. For all systems, the storage modulus (G′) was markedly higher than the loss modulus (G″): G′ increased rapidly and then plateaued over time, whereas G″ remained nearly constant. This behavior confirms the formation of a stable, elasticity-dominated microgel network following structural reconstruction. At 2.0 wt%, the structural reconstruction of the HATA system was completed in ~500 s vs. ~800 s for the DSP-2 system. At 1.0 wt%, the reconstruction time decreased to ~400 s for HATA and ~600 s for DSP-2. Prior to rheological characterization of solutions with different concentrations, adequate static placement for the corresponding duration is required to achieve complete dynamic reconstruction and equilibrium of the microstructure, thus ensuring the reliability and repeatability of test results.

**Figure 6:**
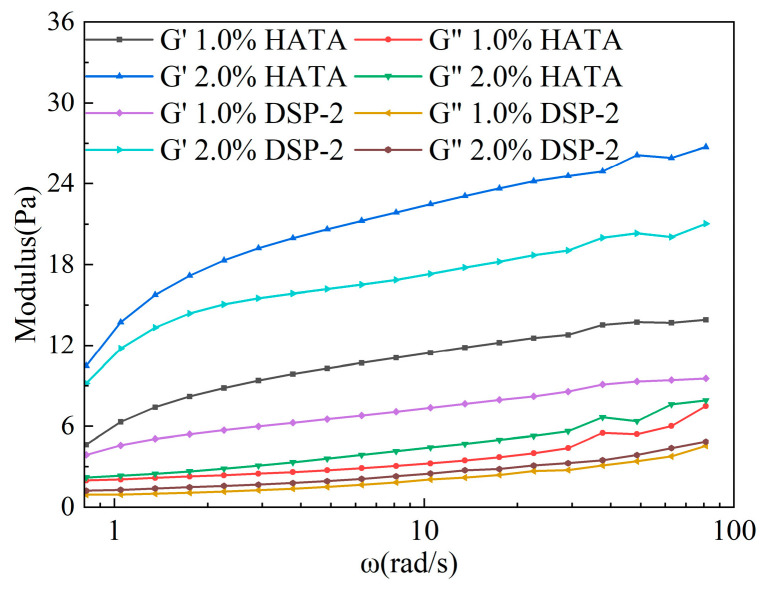
Linear viscoelastic behavior: Effect of concentration on the frequency response of moduli.

The linear viscoelastic frequency sweep results in Figure 6 show that for aqueous HATA and DSP-2 solutions of different concentrations, the storage modulus (G′) was higher than the loss modulus (G″), and both increased with angular frequency, indicating that the systems were elasticity-dominated with stable three-dimensional cross-linked networks formed. Increased concentration significantly enhanced molecular chain entanglement and network strength; at the same concentration, the HATA system exhibited a higher G′/G″ ratio and milder variations in modulus curves with frequency, endowing its network structure with superior stability against perturbations.

**Figure 7:**
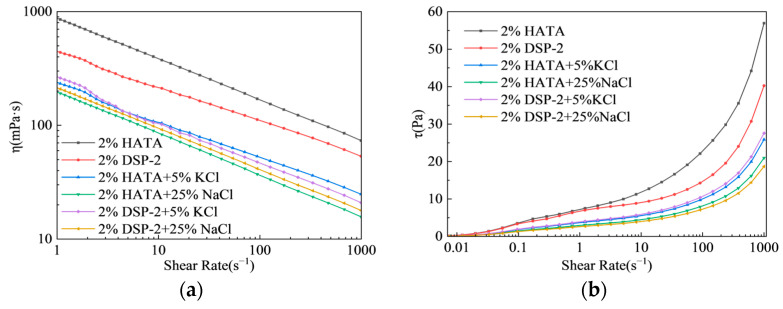
Shear-thinning behavior and stress response: (**a**) Viscosity-shear rate curves; (**b**) Shear stress-shear rate curves.

Figure 7 shows the shear rheology of 2 wt% HATA and DSP-2 aqueous solutions in saline media, where all systems displayed typical shear-thinning behavior. In the salt-free system, 2 wt% HATA had higher initial viscosity and shear stress than 2 wt% DSP-2, indicative of superior thickening performance and network strength. The 2 wt% HATA solution presented a viscosity of 853 mPa·s at 1 s^−1^ and 73 mPa·s at 1000 s^−1^, showing a more significant shear-thinning effect. With the addition of 5 wt% KCl or 25 wt% NaCl, both systems had reduced viscosity and shear stress, while the HATA system exhibited notably higher retention rates of viscosity and shear stress with a milder decline in shear stress. This confirms that HATA molecular chains resist electrolyte-induced electrostatic shielding more effectively in saline environments, thus maintaining a stable 3D entangled polymer network [34,35,36,37].

**Figure 8:**
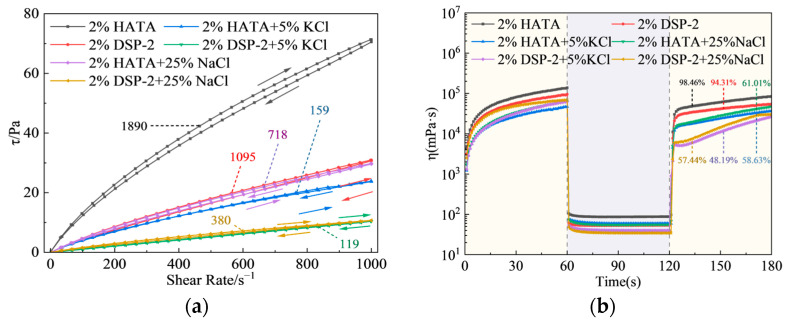
Thixotropy and shear recovery performance: (**a**) shear stress-shear rate hysteresis loop curves; (**b**) viscosity recovery curves with time.

The hysteresis loop areas in Figure 8 show that the neat 2 wt% HATA system had the largest area (2640), significantly higher than that of 2 wt% DSP-2 (1830), indicating that the network structure of HATA undergoes more extensive shear-induced damage and thus exhibits a more pronounced thixotropic response [38,39]. After the addition of 25 wt% NaCl, the loop areas of both systems decreased (2181 for HATA + NaCl and 1312 for DSP-2 + NaCl), demonstrating that salinity impaired the network strength while HATA still maintained a large thixotropic loop area. Viscosity recovery curves (Figure 8b) further show that the 2 wt% HATA system exhibits faster post-shear viscosity recovery, with a recovery efficiency of 98.46%, compared with only 94.31% for the 2 wt% DSP-2 system. Moreover, HATA had better tolerance to salt contamination, with higher recovery degrees under both 5 wt% KCl and 25 wt% NaCl conditions. This property facilitates the rapid gel formation of drilling fluids at rest for cuttings suspension and the quick viscosity recovery after shearing [40,41,42].

### 3.3. Performance Evaluation of HATA in Drilling Fluids

#### 3.3.1. Evaluation of Viscosifying Performance

**Figure 9:**
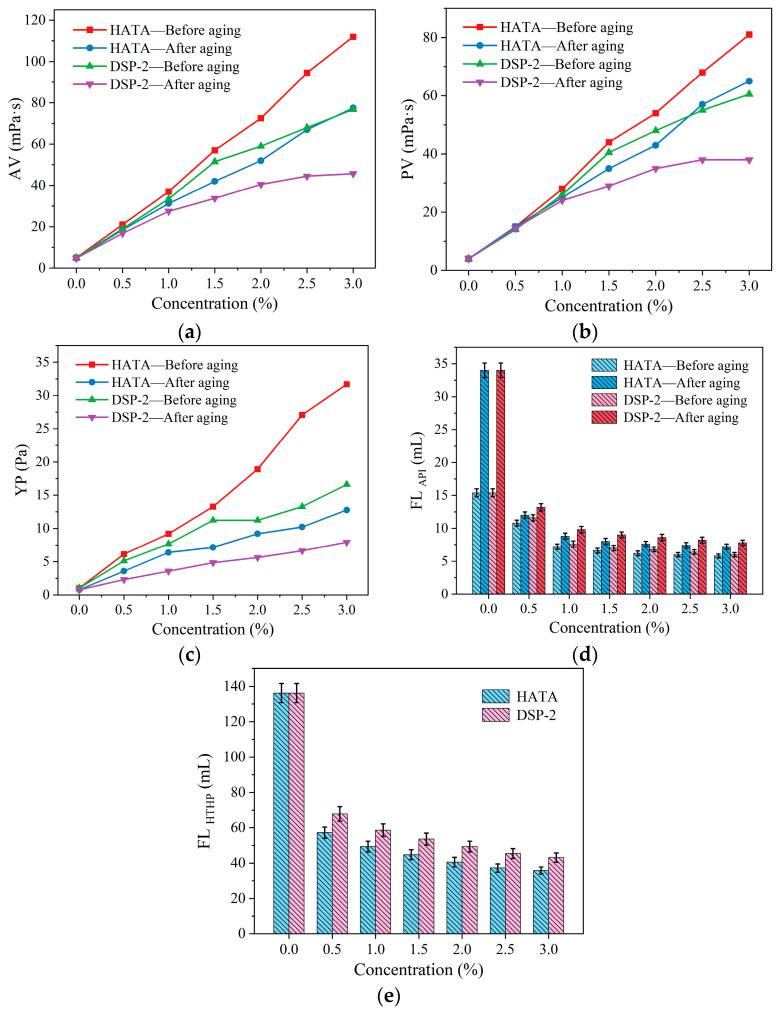
Effect of HATA concentration on the performance of drilling fluid: (**a**) AV; (**b**) PV; (**c**) YP; (**d**) FL_API_; (**e**) FL_HTHP_.

Different concentrations of HATA were incorporated into drilling fluid samples to prepare corresponding formulations. Their rheological properties and fluid loss were assessed both before and after aging at 180 °C, and compared with those of DSP-2. The addition levels of HATA and DSP-2 were 0 wt%, 0.5 wt%, 1.0 wt%, 1.5 wt%, 2.0 wt%, 2.5 wt%, and 3.0 wt%, respectively. The results are shown in Figure 9.

With increasing concentration of HATA, the apparent viscosity (AV), plastic viscosity (PV), and yield point (YP) of the drilling fluid, both before and after aging, exhibited a consistent upward trend, while the API fluid loss and HTHP fluid loss showed a decreasing trend. At a HATA concentration of 3 wt%, the AV, PV, and YP of the drilling fluid before aging reached 112.0 mPa·s, 81.0 mPa·s, and 31.68 Pa, respectively. After aging at 180 °C for 16 h, these values were maintained at 77.5 mPa·s, 65.0 mPa·s, and 12.78 Pa, respectively, corresponding to an apparent viscosity retention rate of 69.20%. The addition of HATA significantly reduced the fluid loss of the drilling fluid. For the fluid containing 3 wt% HATA, the API fluid loss (at room temperature, 0.7 MPa) before aging was 5.8 mL, which increased only to 7.2 mL after high-temperature aging. The HTHP fluid loss (at 180 °C, 3.5 Mpa) was 35.8 mL, representing a 73.72% reduction compared to the drilling fluid without HATA, indicating that the enhanced viscosity effectively controlled fluid loss. In contrast, the drilling fluid containing DSP-2 showed relatively lower increases in AV, PV, and YP, along with higher API and HTHP fluid losses. For the fluid with 3 wt% DSP-2, the apparent viscosity before aging was 76.75 mPa·s, which decreased to 45.75 mPa·s after aging at 180 °C for 16 h, yielding a viscosity retention rate of 59.61%. High-temperature aging increased the API fluid loss from 5.8 mL to 7.9 mL, while the HTHP fluid loss (at 180 °C, 3.5 Mpa) reached 43.2 mL. At the same concentration, HATA outperformed DSP-2 in enhancing viscosity and yield point, as well as in controlling fluid loss, demonstrating enhanced thickening and filtration control performance relative to DSP-2.

#### 3.3.2. Evaluation of Thermal Performance

**Figure 10:**
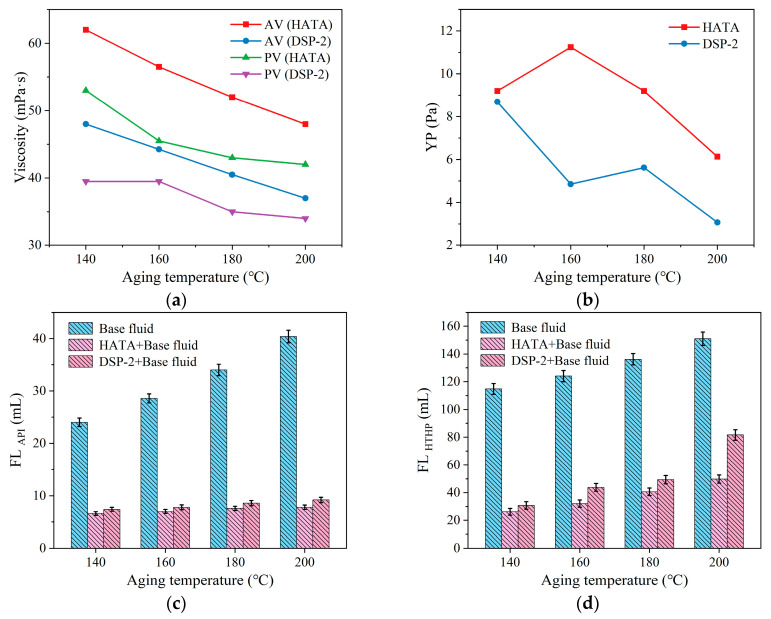
Effect of aging temperature on the performance of drilling fluid: (**a**) viscosity; (**b**) YP; (**c**) FL_API_; (**d**) FL_HTHP_.

Add 2 wt% HATA and DSP-2, respectively, to the drilling fluid to prepare corresponding samples, then test their rheological properties and fluid loss. Subsequently, subject them to aging at different temperatures, cool to room temperature, and retest their rheological properties and fluid loss. The hot rolling temperatures were 140 °C, 160 °C, 180 °C, and 200 °C, with an aging duration of 16 h. The test results are shown in Figure 10.

Elevating the aging temperature caused a continuous decrease in AV and PV for all drilling fluid formulations, with a concomitant steady increase in API and HTHP filtration loss. As shown in Figure 10, at an aging temperature of 200 °C, the drilling fluid containing 2 wt% HATA exhibited an apparent viscosity of 48.0 mPa·s, a plastic viscosity of 42.0 mPa·s, and a yield point of 6.13 Pa. The 2 wt% DSP-2-containing fluid recorded values of 37.0 mPa·s, 34.0 mPa·s, and 3.07 Pa. Regarding filtrate loss control, after aging at 200 °C, the API filtrate loss of the drilling fluid containing 2 wt% HATA was 7.8 mL, while the HTHP filtrate loss (200 °C, 3.5 MPa) was 53.8 mL. Under identical conditions, the drilling fluid containing DSP-2 exhibited an API loss of 9.2 mL and an HTHP loss of 64.6 mL. Comparative analysis revealed that HATA demonstrated superior thermal stability compared to DSP-2. At 200 °C, it effectively adsorbed bentonite particles to maintain the drilling fluid’s internal spatial structure, thereby preserving viscosity and yield point while controlling fluid loss.

**Figure 11:**
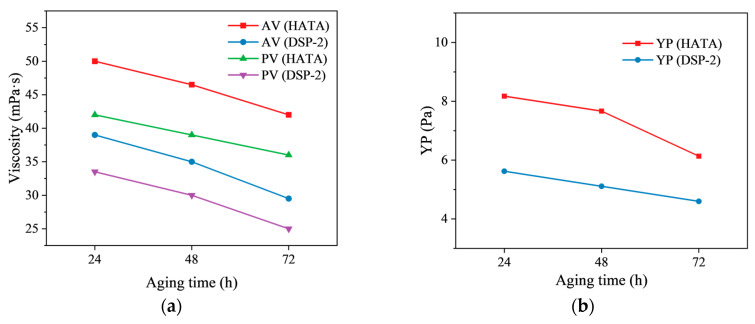
Effect of aging time on the performance of drilling fluid: (**a**) viscosity; (**b**) YP.

To investigate the long-term thermal stability of drilling fluids, samples containing 2 wt% HATA and DSP-2 were hot-rolled at 200 °C for 24, 48, and 72 h. After cooling, their rheological properties were tested, and the results are shown in Figure 11. With increasing aging time, the apparent viscosity (AV), plastic viscosity (PV), and yield point (YP) of both systems gradually decreased. After aging for 24 h, the apparent viscosity and yield point of the HATA-based fluid were 50 mPa·s and 8.176 Pa, respectively, which decreased to 42 mPa·s and 6.132 Pa after 72 h. After 72 h of aging, the rheological parameters of the DSP-2-based fluid decreased more significantly, with the apparent viscosity and yield point falling to 29.5 mPa·s and 4.599 Pa, respectively. The HATA-containing drilling fluid maintained higher rheological parameters throughout the aging process, exhibiting superior long-term thermal stability.

#### 3.3.3. Evaluation of Salt Tolerance

**Figure 12:**
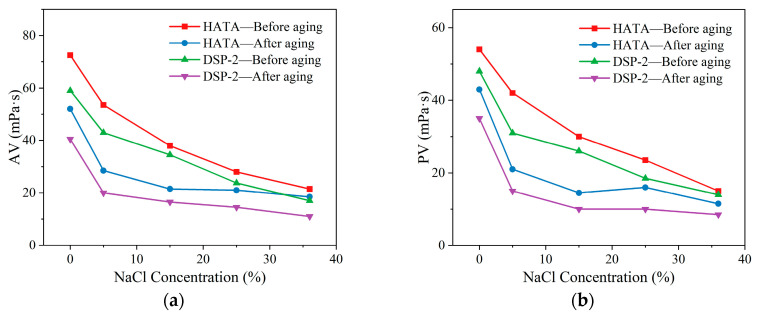

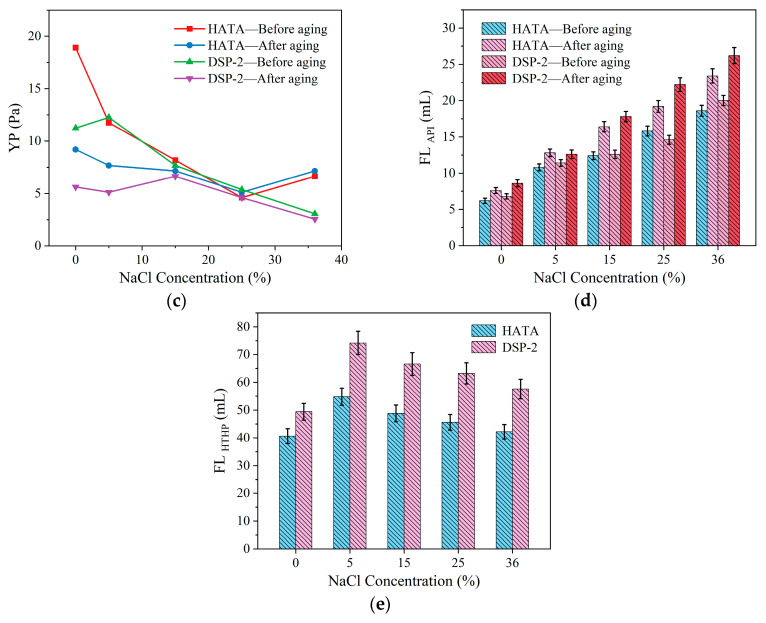
Effect of NaCl concentration on the performance of drilling fluid: (**a**) AV; (**b**) PV; (**c**) YP; (**d**) FL_API_; (**e**) FL_HTHP_.

To prepare corresponding brine drilling fluid samples, 2 wt% HATA and DSP-2 were added to the drilling fluid respectively, followed by varying quantities of NaCl. Rheological properties and filtrate loss were tested both before and after aging at 180 °C, and compared with those of the DSP-2-based fluid. NaCl was added at concentrations of 0 wt%, 5 wt%, 15 wt%, 25 wt%, and 36 wt%. The test results are presented in Figure 12.

As the NaCl concentration increased, the viscosity of all unaged drilling fluid samples exhibited a decreasing trend. HATA-modified drilling fluids exhibited a slower rate of viscosity reduction, with a modest increase in API and HTHP filtration loss relative to DSP-2-modified fluids. Under high-salinity conditions containing 36 wt% NaCl, the drilling fluid with 2 wt% HATA exhibited an apparent viscosity of 21.5 mPa·s, which remained at 18.5 mPa·s after aging, yielding an apparent viscosity retention rate of 86.05%. The drilling fluid containing 2 wt% DSP-2 exhibited an apparent viscosity of 17.0 mPa·s, which decreased to 11.0 mPa·s after aging, representing a viscosity retention rate of 64.71%. Regarding filtrate loss control, under high-salinity conditions containing 36 wt% NaCl, the API filtrate loss of the drilling fluid with 2 wt% HATA after aging was 23.4 mL, and the HTHP filtrate loss was 42.2 mL, both lower than those of the drilling fluid containing 2 wt% DSP-2. The rheological stability and fluid loss control efficacy in high-salinity environments stem from HATA’s hydrophobic association network, which maintains stable hydration and intermolecular structure under high mineralization conditions. Conversely, DSP-2 readily loses performance due to salt ion shielding, failing to sustain adequate viscosity enhancement.

### 3.4. Mechanism Analysis of HATA

#### 3.4.1. Zeta Potential Analysis

**Figure 13:**
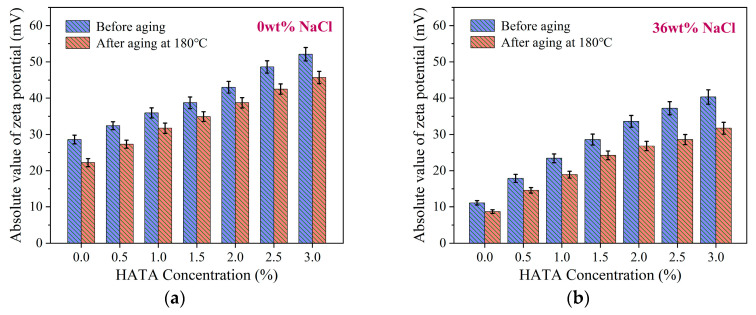
Analysis of the absolute value of zeta potential: (**a**) absolute value of zeta potential at 0 wt% NaCl; (**b**) absolute value of zeta potential at 36 wt% NaCl.

Drilling fluid constitutes a colloidal dispersion system of bentonite and water, whose stability can be evaluated through zeta potential testing. The absolute zeta potential magnitude directly reflects the anti-agglomeration capacity of the drilling fluid colloidal system: an absolute zeta potential > 35 mV is widely accepted as the threshold for a stable colloidal dispersion in drilling fluids. This measurement also assesses the extent to which high-temperature and high-salinity conditions impact system stability. HATA was added at varying concentrations to prepared drilling fluids. Zeta potentials were measured under 0 wt% NaCl and 36 wt% NaCl conditions, followed by retesting after 16 h of hot rolling at 180 °C. HATA concentrations tested were 0 wt%, 0.5 wt%, 1.0 wt%, 1.5 wt%, 2.0 wt%, 2.5 wt%, and 3.0 wt%. The test results are presented in Figure 13.

The absolute value of the drilling fluid’s zeta potential increases with rising HATA concentration. At 3 wt% HATA, the absolute zeta potential reaches 52.1 mV, decreasing to 45.7 mV after aging at 180 °C. In a 36 wt% NaCl brine-based mud, 3 wt% HATA elevated the absolute zeta potential to 40.3 mV, which decreased to 31.7 mV after aging at 180 °C. Following high-temperature aging, the absolute zeta potential of the HATA system remained at a high level in both freshwater and high-salinity environments. This indicates that HATA enhances the stability of colloidal dispersion systems by adsorbing onto bentonite particle surfaces, strengthening their hydration, and increasing surface negative charge, thereby counteracting the adverse effects of high-temperature, high-salinity environments on drilling fluids [43,44].

#### 3.4.2. Adsorption Capacity Analysis

**Figure 14:**
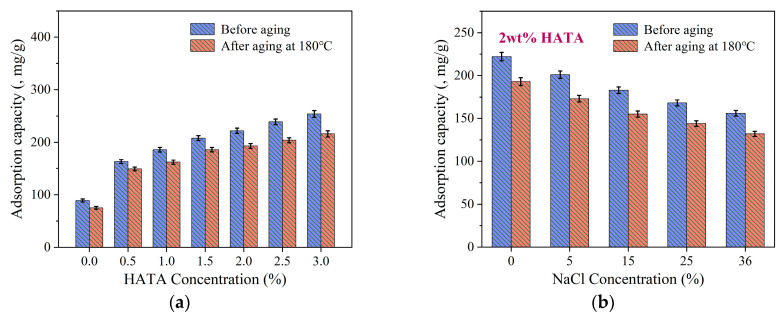
Adsorption capacity analysis: (**a**) adsorption variation with HATA concentration and (**b**) adsorption variation at different NaCl concentrations.

To evaluate the adsorption behavior of HATA on bentonite surfaces, the TOC adsorption capacities were measured at different HATA dosages and NaCl concentrations, and their variation rules after hot rolling aging at 180 °C were investigated. As shown in Figure 14, the results showed that with the increase in HATA dosage from 0 wt% to 3.0 wt%, the adsorption capacity of bentonite for HATA increased progressively, rising from 89 mg/g to 254 mg/g before aging; it decreased slightly after aging at 180 °C but still remained at a high level of approximately 216 mg/g at 3.0 wt% HATA, demonstrating excellent high-temperature resistant adsorption stability. At a fixed HATA dosage of 2 wt%, the adsorption capacity decreased gradually with the increase in NaCl concentration from 0 wt% to 36 wt%, dropping from about 222 mg/g to 156 mg/g before aging, and still maintained a considerable level of about 132 mg/g under 36 wt% high salinity after aging at 180 °C. This indicated that although a high-salinity environment exhibited a certain inhibitory effect on adsorption, HATA could still retain strong adsorption activity. The above results confirmed that HATA can effectively resist the adverse effects of high-temperature and high-salinity environments on the colloidal dispersion system of drilling fluid through stable adsorption on bentonite surfaces.

#### 3.4.3. Particle Size Distribution Analysis

**Figure 15:**
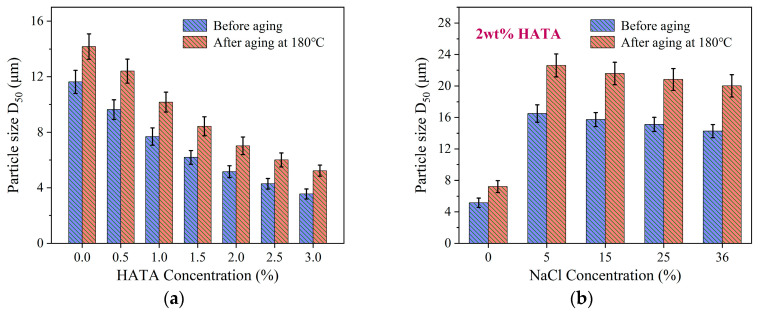
Analysis of drilling fluid particle size distribution: (**a**) particle size distribution of drilling fluids with varying HATA concentrations; (**b**) particle size distribution of drilling fluids at different NaCl concentrations.

Add HATA at varying concentrations to the drilling fluid to prepare corresponding drilling fluid samples. Maintain the HATA content at 2 wt% while adding different concentrations of NaCl to prepare corresponding drilling fluid samples. The D_50_ particle size values were measured before and after drilling fluid aging. HATA was added at concentrations of 0, 0.5 wt%, 1.0 wt%, 1.5 wt%, 2.0 wt%, 2.5 wt%, and 3.0 wt%, while NaCl was added at concentrations of 0 wt%, 5 wt%, 15 wt%, 25 wt%, and 36 wt%. The test results are presented in Figure 15.

As shown in Figure 15a, both pre- and post-aging D50 values exhibit a significant decreasing trend as HATA concentration increases from 0 to 3.0 wt%. At 0 wt% HATA, the pre-aging D50 was 11.63 μm, increasing to 14.17 μm post-aging. In contrast, at 3.0 wt% HATA system, the D_50_ decreased to 3.56 μm before aging and remained at 5.23 μm after aging. This observation is attributed to the interfacial adsorption of HATA: its molecular chains increase the negative electrostatic repulsion on bentonite particle surfaces via charge modification, while inhibiting particle agglomeration through molecular bridging. This results in greater dispersion of solid particles within the drilling fluid and a refinement of particle size distribution.

Figure 15b demonstrates that as the NaCl concentration increased from 5 wt% to 36 wt%, the particle size D_50_ remained largely unchanged before and after drilling fluid aging, maintaining a stable range overall. This demonstrates HATA’s exceptional salt dispersion capability. In high-salinity environments, HATA counteracts the compressive effect of Na^+^ on bentonite’s double electric layer through the synergistic interaction of its hydrophobic association structure and multiple hydrophilic groups. This prevents particle agglomeration caused by weakened electrostatic repulsion, thereby maintaining particle dispersion even after high-salinity (36 wt% NaCl) and high-temperature aging.

#### 3.4.4. Microstructural Analysis

Drilling fluid samples were prepared by adding varying concentrations of HATA and NaCl to the drilling fluid. These samples were subjected to hot rolling at 180 °C for 16 h, followed by API filtration loss testing. The resulting mud cake was freeze-dried, and its microstructure was examined using an EVO-15/LS scanning electron microscope. HATA was added at concentrations of 0 wt% and 2.0 wt%, while NaCl was added at concentrations of 0 wt%, 15 wt%, and 36 wt%.

**Figure 16:**
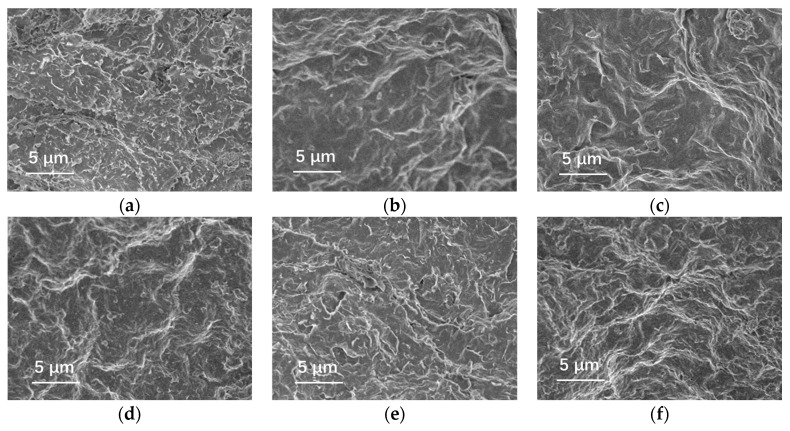
Microscopic morphological analysis of drilling fluid mud cake: (**a**) drilling fluid (**b**) drilling fluid + 15 wt% NaCl, (**c**) drilling fluid + 36 wt% NaCl, (**d**) drilling fluid + 2 wt% HATA, (**e**) drilling fluid + 2 wt% HATA + 15 wt% NaCl, (**f**) drilling fluid + 2 wt% HATA + 36 wt% NaCl.

Figure 16a shows a loose, porous mud cake structure with low agglomeration between solid particles. The flocculent/fibrous structure exhibits poor continuity, featuring large pore sizes and uneven distribution. In this state, the mud cake demonstrates weak filtration loss control, with insufficient network framework formation capability from the base mud’s solid particles. Figure 16b shows that the ionic action of sodium chloride compresses the double electric layer of particles, enhancing particle aggregation. The mud cake structure is denser than in Figure 16a, yet numerous pores remain, and structural uniformity is generally poor. In Figure 16c, the double electric layer is further compressed at medium-high salinity, resulting in more pronounced particle aggregation and a denser mud cake structure. However, localized excessive particle clustering occurs, reducing structural uniformity. While high salinity enhances particle aggregation, excessive clustering readily leads to mud cake heterogeneity characterized by ‘localized compaction and localized loosening’.

In the mud cake images showing HATA addition, the mud cake transformed from a loose, porous structure to a dense network structure following viscosifier addition, with significant optimization in pore size and distribution. In Figure 16d, the macromolecular chains of the viscosifier formed a continuous fibrous/flocculent network within the mud cake, tightly bridging solid particles. This resulted in a markedly denser cake structure with substantially reduced pore size and uniform distribution, demonstrating its reinforcing effect on the mud cake framework. In Figure 16e, at 15% sodium chloride salinity, the viscosifier’s network structure maintained good compactness and uniformity, with ionic interactions failing to notably disrupt the macromolecular bridging. This indicates HATA exhibits favorable salt stability at moderate salinity, synergistically leveraging both ionic aggregation and macromolecular bridging to yield a superior cake structure compared to the unsalted control group at equivalent salinity. Figure 16f reveals that although the extensibility of HATA macromolecular chains slightly decreased under high salinity, a uniform network structure was still formed, significantly outperforming the control group without a viscosifier at the same salinity. This demonstrates that the viscosifier remains effective in high-salinity environments and exhibits favorable salt tolerance.

**Figure 17:**
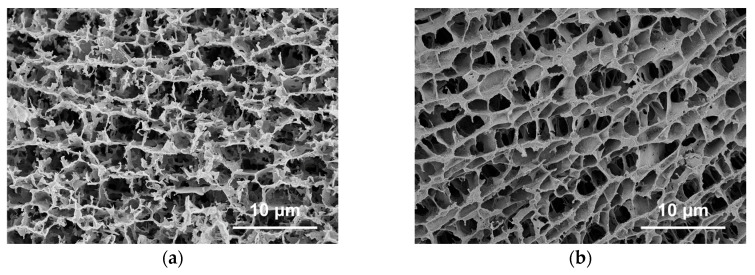
Microstructure analysis of drilling fluid: (**a**) drilling fluid; (**b**) drilling fluid + 2 wt% HATA.

The colloidal microstructures of the base mud and drilling fluid with 2 wt% HATA after aging at 180 °C were observed via cryo-scanning electron microscopy, as shown in Figure 17. After high-temperature aging at 180 °C, bentonite particles in the base mud exhibited a loose network with small, unevenly distributed pores, which was attributed to the deteriorated microstructure caused by weakened hydration and unbalanced interparticle forces of the colloidal system under high temperature. Under the same aging conditions, the drilling fluid with 2 wt% HATA formed a more regular, uniform three-dimensional network with larger pores between bentonite particles, featuring more stable interparticle connections and improved dispersion state. This morphological difference indicates that HATA adsorbs on the surface of bentonite particles to strengthen the hydration film and electrostatic repulsion of particles, which effectively resists the damage of high-temperature aging to the colloidal microstructure and maintains the structural stability of the drilling fluid dispersion system, being well consistent with the test results of zeta potential and TOC adsorption capacity.

#### 3.4.5. Mechanism of HATA

The superior salt tolerance, thermal degradation resistance, colloidal stabilization, and viscosifying effect of HATA in drilling fluids are attributed to the synergy between its molecular structure—copolymerized from AM, AMPS, SSS, and SMA monomers—and the colloidal characteristics of the drilling fluid system. The underlying mechanism can be summarized as follows:

Colloidal stability regulation is the core mechanism by which HATA maintains essential drilling fluid performance [45,46]. The diffuse double layer generated by the negative charges on bentonite particles—the primary dispersed phase—is crucial for colloidal stability. In high-salinity environments, Na^+^ compresses this layer, inducing particle aggregation and system instability when the zeta potential falls to ≤10 mV. HATA adsorbs onto bentonite surfaces via hydrogen bonding between its amide groups and clay hydroxyls. Simultaneously, its sulfonate groups introduce additional negative charges, increasing surface charge density, expanding the double layer, and enhancing interparticle electrostatic repulsion. This effectively offsets the compression effect of salt ions [47,48]. Experimental results confirm this mechanism: in a 0 wt% NaCl system, increasing HATA concentration from 0 to 3 wt% raised the absolute zeta potential from 28.6 mV to 52.1 mV before aging. Even in 36 wt% NaCl, the system with 3 wt% HATA retained a zeta potential of −31.7 mV after aging at 180 °C, a 316% increase over the additive-free system (7.62 mV). These results demonstrate HATA’s effectiveness in mitigating salt-induced colloidal destabilization via adsorption and charge regulation.

HATA’s viscosifying ability stems from its hydrophobic long-chain alkyl and micro-crosslinking units, which promote intermolecular association and entanglement. Hydrophobic groups form dynamic physical crosslinks under the salting-out effect of ions, while polymer chains adsorbed on different bentonite particles bridge them together. Combined with chain entanglement, this leads to a stable three-dimensional network [49,50]. This structure significantly increases the apparent viscosity (AV) and yield point (YP), imparting shear-thinning behavior (reducing circulation friction under high shear) and rapid structural recovery once shear decreases (maintaining cuttings suspension capacity) [51].

The molecular structural stability constitutes the fundamental reason for HATA’s tolerance to extreme high-temperature and high-salinity conditions. The HATA molecular backbone employs carbon-carbon bonds with a bond energy as high as 347 kJ/mol. Concurrently, copolymerization introduces sodium styrenesulfonate monomers containing rigid benzene rings, significantly enhancing the backbone’s resistance to thermal degradation [52]. The incorporation of 2-acrylamido-2-methylpropane sulfonic acid (AMPS) units, with their strongly hydrophilic sulfonic acid groups, forms a stable hydration layer. This prevents hydrophobic collapse and aggregation of the molecular chains at elevated temperatures [53]. This synergistic effect of the ‘rigid backbone-stable hydration layer’ enables HATA to maintain molecular structural integrity and functional efficacy under combined high-temperature and high-salinity stresses.

**Figure 18:**
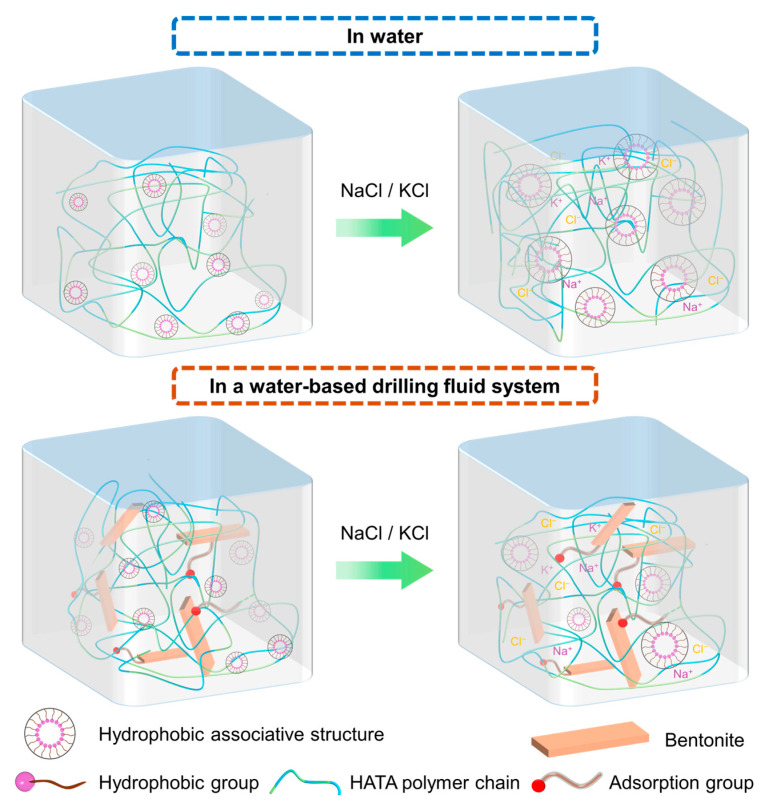
Schematic diagram of hydrophobic association and viscosity enhancement mechanism of HATA in water and drilling fluid.

As shown in Figure 18, HATA enhances drilling fluid performance through charge regulation, network formation, and structural stability. Adsorption and charge modification impart colloidal stability; hydrophobic association and particle bridging build a viscosity-enhancing network; while a rigid backbone and hydrophilic groups ensure tolerance in harsh environments. This mechanistic alignment between molecular design and functional requirements provides a theoretical foundation for developing high-performance additives for high-temperature, high-salinity applications.

## 4. Conclusions

To address the insufficient thermal and salt tolerance of conventional viscosifiers for deep and ultra-deep water-based drilling fluids, a hydrophobic associative polymer viscosifier, HATA, was synthesized, and its structural characterization, performance evaluation, and action mechanism were investigated. The main conclusions are as follows:

The quaternary hydrophobic associative polymer HATA was successfully synthesized, with an initial thermal decomposition temperature of 230.6 °C, a weight-average molecular weight of 3,449,076 g/mol, and a polydispersity index of 2.08. Its ultra-high molecular weight and excellent thermal stability provide a structural basis for superior viscosifying performance.

HATA demonstrates enhanced thickening and filtration control performance in bentonite-based slurries. With 3 wt% HATA added to 4 wt% base slurry, the apparent viscosity reached 112.0 mPa·s at room temperature and remained at 77.5 mPa·s after hot rolling at 180 °C for 16 h. The API filtration loss was 7.2 mL, and the HTHP filtration loss was reduced by 73.72% compared with the blank system, significantly outperforming the commercial viscosifier DSP-2.

HATA shows excellent thermal stability. After hot rolling at 200 °C for 16 h, the 2 wt% HATA-modified base slurry still maintained an apparent viscosity of 48.0 mPa·s and a yield point of 6.13 Pa. Even after 72 h at 200 °C, its rheological parameters were considerably higher than those of the DSP-2-modified system.

HATA possesses superior salt tolerance. In 36 wt% NaCl solution, the 2 wt% HATA-modified slurry retained 86.05% of its apparent viscosity after aging at 180 °C, with API and HTHP filtration losses of 23.4 mL and 42.2 mL, respectively, far better than the DSP-2 system under the same conditions.

HATA sustains stable rheological and filtration performance under high-temperature and high-salinity conditions via a threefold synergistic mechanism: electrostatic charge regulation, network formation, and molecular structural stabilization. It enhances colloidal stability, delivers efficient viscosity enhancement, and resists thermal degradation and salt interference. As a high-temperature and high-salinity-tolerant viscosifier for WBDFs, HATA offers a technical alternative for drilling fluid formulation optimization in deep oil and gas exploration.

## Figures and Tables

**Table 1:** The results of the relative molecular weight of HATA.

M_w_ g/mol	M_n_ g/mol	M_z_ g/mol	M_p_ g/mol	M_z+1_ g/mol	PD Mw/Mn
3,449,076	1,658,370	13,512,573	1,996,483	28,350,629	2.08

## Data Availability

The data supporting the findings of this study are available within the article. Further inquiries should be directed to the corresponding author.

## References

[1] Karakosta K., Mitropoulos A.C., Kyzas G.Z. (2021). A review in nanopolymers for drilling fluids applications. J. Mol. Struct..

[2] Sun J., Yang J., Rong K., Wang R., Qu Y., Liu F. (2023). Advances in study on rheology modifier for water-based drilling fluids. Xinjiang Oil Gas.

[3] Sun J., Yang J., Lv K., Bai Y., Liu J., Huang X. (2025). Research status and prospect of deep and ultra-deep drilling technology. Xinjiang Oil Gas.

[4] Liu F., Sun J., Wang J. (2023). A global review of technical status and development trend of drilling fluids for deep and ultra-deep wells. Xinjiang Oil Gas.

[5] Balaga D.K., Mondal J., Tatke V., Bandal R., Kulkarni S.D. (2023). Degradation kinetics of synthetic acrylamide polymers as a viscosity and filtration control additives in the low-density water–based drilling fluid systems. Geoenergy Sci. Eng..

[6] Wang X., Wang G., Wang Y., Wan S., Wang L., Xu H. (2025). Development and application of a high temperature environmentally friendly drilling fluid system. Xinjiang Oil Gas.

[7] Wang Y., Du Y., Yang Z., Zou J., Fan M., Zhang X. (2024). Effects of polymers with different anionic groups in drilling fluids and their properties of temperature and salt tolerance. J. Macromol. Sci. Part A Pure Appl. Chem..

[8] Sepehri S., Soleyman R., Varamesh A., Valizadeh M., Nasiri A. (2018). Effect of synthetic water-soluble polymers on the properties of the heavy water-based drilling fluid at high pressure-high temperature (HPHT) conditions. J. Pet. Sci. Eng..

[9] Xie B., Liu X., Wang H., Zheng L. (2016). Synthesis and application of sodium 2-acrylamido-2-methylpropane sulphonate/N-vinylcaprolactam/divinyl benzene as a high-performance viscosifier in water-based drilling fluid. J. Appl. Polym. Sci..

[10] Shanmugam S., Ross G., Mbuncha C.Y., Santra A. (2021). Rapid, green synthesis of high performance viscosifiers via a photoiniferter approach for water-based drilling fluids. Polym. Chem..

[11] Quitian-Ardila L.H., Garcia-Blanco Y.J., Daza-Barranco L.M., Schimicoscki R.S., Andrade D.E.V., Franco A.T. (2024). Improving the rheological and thermal stability of water-based drilling fluids by incrementing xanthan gum concentration. Phys. Fluids.

[12] Akpan E.U. (2024). Utilizing environmentally friendly polymers as rheological control and fluid loss additives in water-based drilling muds. Geoenergy Sci. Eng..

[13] Villada Y., Gallardo F., Erdmann E., Casis N., Olivares L., Estenoz D. (2017). Functional characterization on colloidal suspensions containing xanthan gum (XGD) and polyanionic cellulose (PAC) used in drilling fluids for a shale formation. Appl. Clay Sci..

[14] Ali I., Ahmad M., Lashari N. (2024). Optimizing filtration properties of water based drilling mud systems using dually modified starch. J. Clean. Prod..

[15] Oseh J.O., Mohd N.M.N.A., Gbadamosi A.O., Agi A., Blkoor S.O., Ismail I., Igwilo K.C., Igbafe A.I. (2023). Polymer nanocomposites application in drilling fluids: A review. Geoenergy Sci. Eng..

[16] Villada Y., Iglesias M.C., Casis N., Erdmann E., Peresin M.S., Estenoz D. (2018). Cellulose nanofibrils as a replacement for xanthan gum (XGD) in water based muds (WBMs) to be used in shale formations. Cellulose.

[17] Zhou G., Zhang X., Yan W., Qiu Z. (2025). Synthesis, characteristics, and field applications of high-temperature and salt-resistant polymer gel tackifier. Gels.

[18] Zhao H., Liu D., Kang W., Wu D., Dong Y., Wang J., Yang H., Sarsenbekuly B. (2024). Salt thickening performance and mechanism of an N-Vinyl-2-Pyrrolidinone based amphiphilic polymer. J. Mol. Liq..

[19] Misbah B., Sedaghat A., Balhasan S., Elgaddafi R., Malayer M.A., Malhas R.N., Omar M., Benomran M. (2023). Enhancing thermal stability and filtration control for water-based drilling fluid using viscosifier polymers and potassium chloride additives. Geoenergy Sci. Eng..

[20] Cao P.-F., Mangadlao J.D., Advincula R.C. (2015). Stimuli-responsive polymers and their potential applications in oil-gas industry. Polym. Rev..

[21] Chu Q., Lin L. (2019). Synthesis and properties of an improved agent with restricted viscosity and shearing strength in water-based drilling fluid. J. Pet. Sci. Eng..

[22] Xie B., Ting L., Zhang Y., Liu C. (2018). Rheological properties of bentonite-free water-based drilling fluids with novel polymer viscosifier. J. Pet. Sci. Eng..

[23] Wang H., Li M., Qin X., Sun K., Zhang C. (2022). Hydrophobic association copolymer as viscosifier for high-temperature high-pressure anti-sedimentation performance of water-based drilling fluid. Polym. Eng. Sci..

[24] Albrecht M., Rice C.A., Suhm M.A. (2008). Elementary peptide motifs in the gas phase: FTIR aggregation study of formamide, acetamide, N-methylformamide, and N-methylacetamide. J. Phys. Chem. A.

[25] Khoshnood N., Shahrezayee M.H., Shahrezayee M., Shams A., Zamanian A. (2022). Biological study of polyethyleneimine functionalized polycaprolactone 3D-printed scaffolds for bone tissue engineering. J. Appl. Polym. Sci..

[26] Tevlek A., Agacik D.T., Aydin H.M. (2020). Stretchable poly(glycerol-sebacate)/β-tricalcium phosphate composites with shape recovery feature by extrusion. J. Appl. Polym. Sci..

[27] Patra A.S., Ghorai S., Ghosh S., Mandal B., Pal S. (2016). Selective removal of toxic anionic dyes using a novel nanocomposite derived from cationically modified guar gum and silica nanoparticles. J. Hazard. Mater..

[28] Dalla Valle C., Zecca M., Rastrelli F., Tubaro C., Centomo P. (2020). Effect of the sulfonation on the swollen state morphology of styrenic cross-linked polymers. Polymers.

[29] Sharma P., Pandey O.P., Diwan P.K. (2019). Non-isothermal kinetics of pseudo-components of waste biomass. Fuel.

[30] Ceretti D.V.A., Edeleva M., Cardon L., D’hooge D.R. (2023). Molecular pathways for polymer degradation during conventional processing, additive manufacturing, and mechanical recycling. Molecules.

[31] Gikarakis T., Pappas I., Arvanitaki P., Pantazi E., Mitsoni E., Roka N., Pitsikalis M. (2021). Thermal stability and kinetics of thermal decomposition of statistical copolymers of N-vinylpyrrolidone and alkyl methacrylates synthesized via RAFT polymerization. J. Chem..

[32] Gissinger J.R., Zavada S.R., Smith J.G., Kemppainen J., Gallegos I., Odegard G.M., Siochi E.J., Wise K.E. (2023). Predicting char yield of high-temperature resins. Carbon.

[33] Yang X., Li Y., Lei W., Liu X., Zeng Q., Liu Q., Feng W., Li K., Wang P. (2021). Thermal degradation behaviors of poly (arylene ether nitrile) bearing pendant carboxyl groups. Polym. Degrad. Stab..

[34] Huff C., Biehler E., Quach Q., Long J.M., Abdel-Fattah T.M. (2021). Synthesis of highly dispersive platinum nanoparticles and their application in a hydrogen generation reaction. Colloids Surf. A.

[35] Zheng H., Shi Y., Tian Y., Yan X., Chen Q., Wu A., An F., Li Y. (2025). Preparation and performance evaluation of non-chemically crosslinked four-tailed hydrophobic associative polymer thickeners. Colloids Surf. A.

[36] Chen N., Lee Y.M. (2021). Anion exchange polyelectrolytes for membranes and ionomers. Prog. Polym. Sci..

[37] Fashandi M., Karamikamkar S., Leung S.N., Naguib H.E., Hong J., Liang B., Park C.B. (2022). Synthesis, structures and properties of hydrophobic Alkyltrimethoxysilane-Polyvinyltrimethoxysilane hybrid aerogels with different alkyl chain lengths. J. Colloid Interface Sci..

[38] Fijneman A.J., Heinrichs J.M.J.J., van Leuken S.H.M., de With G., Friedrich H. (2021). Time-resolved investigation of mesoporous silica microsphere formation using in situ heating optical microscopy. J. Colloid Interface Sci..

[39] Ma S., Xu M., Zhao Z., Pan J., Zhao S., Xue J., Ye Z. (2022). Preparation of 3D superhydrophobic porous g-C3N4 nanosheets@carbonized kapok fiber composites for oil/water separation and treating organic pollutants. Colloids Surf. A.

[40] Gulraiz S., Gray K.E. (2020). Thixotropy effects on drilling hydraulics. J. Nat. Gas Sci. Eng..

[41] Rodrigues T., Galindo-Rosales F.J., Campo-Deaño L. (2020). Haemodynamics around confined microscopic cylinders. J. Non-Newton. Fluid Mech..

[42] Yang T., Wang J., Qiao D., Li G., Cheng H., Zhang X., Shi R. (2024). The thixotropic characteristics of rheological parameters of waste stone-tailings cemented paste backfill and the time effect of pipeline resistance. Constr. Build. Mater..

[43] Siegnin R., Dedzo G.K., Ngameni E. (2022). Sulfonation of the interlayer surface of kaolinite. Appl. Clay Sci..

[44] Shu N.-K., Xu Z.-C., Gong Q.-T., Ma W.-J., Zhang L., Zhang L. (2021). Effect of electrolyte on the surface dilational rheological properties of branched cationic surfactant. Colloids Surf. A.

[45] Ramsis Y., Gravanis E., Sarris E. (2024). Colloidal properties of polymer amended Cyprus bentonite for water-based drilling fluid applications. Colloids Surf. A.

[46] Lv K., Du H., Sun J., Huang X., Shen H. (2022). A thermal-responsive zwitterionic polymer gel as a filtrate reducer for water-based drilling fluids. Gels.

[47] Brahana P.J., Bharti B. (2025). Water salinity impacts aggregation, settling, and deposition of fluvial sediment. ACS Environ. Au.

[48] Li L., Wang L., Liu Q. (2023). Effects of salinity and pH on clay colloid aggregation in ion-adsorption-type rare earth ore suspensions by light scattering analysis. Minerals.

[49] Wong C.K., Qiang X., Müller A.H.E., Gröschel A.H. (2020). Self-Assembly of block copolymers into internally ordered microparticles. Prog. Polym. Sci..

[50] Xiang Y., Tu Z., Lei T., Zhang J., Yeh J. (2021). Multiple-step drawing innovative ultrahigh-molecular-weight polyethylene fibers modified with bacterial cellulose and scCO_2_-aid. J. Appl. Polym. Sci..

[51] Sun J., Zhang X., Lv K., Liu J., Xiu Z., Wang Z., Huang X., Bai Y., Wang J., Jin J. (2022). Synthesis of hydrophobic associative polymers to improve the rheological and filtration performance of drilling fluids under high temperature and high salinity conditions. J. Pet. Sci. Eng..

[52] Ma J., Xia B., Yu P., An Y. (2020). Comparison of an emulsion- and solution-prepared acrylamide/AMPS copolymer for a fluid loss agent in drilling fluid. ACS Omega.

[53] Li J., Ji Y., Ni X., Lv K., Huang X., Sun J. (2024). A micro-crosslinked amphoteric hydrophobic association copolymer as high temperature- and salt-resistance fluid loss reducer for water-based drilling fluids. Pet. Sci..

